# Metal-Induced Production of a Novel Bioadsorbent Exopolysaccharide in a Native *Rhodotorula mucilaginosa* from the Mexican Northeastern Region

**DOI:** 10.1371/journal.pone.0148430

**Published:** 2016-02-01

**Authors:** Maria Teresa Gonzalez Garza, Daniel Barboza Perez, Augusto Vazquez Rodriguez, Domingo Ixcoatl Garcia-Gutierrez, Xristo Zarate, Maria Elena Cantú Cardenas, Ludwing Ilytch Urraca-Botello, Ulrico Javier Lopez-Chuken, Alberto Ludovico Trevino-Torres, Felipe de Jesus Cerino-Córdoba, Pavel Medina-Ruiz, Juan Francisco Villarreal-Chiu, Jose Ruben Morones-Ramirez

**Affiliations:** 1 Facultad de Ciencias Químicas, Universidad Autónoma de Nuevo León, Pedro de Alba, S/N, San Nicolás de los Garza, Nuevo León, México; 2 Facultad de Ingeniería Mecánica y Eléctrica, Universidad Autónoma de Nuevo León, Pedro de Alba, S/N, San Nicolás de los Garza, Nuevo León, México; 3 Facultad de Ciencias Biológicas, Universidad Autónoma de Nuevo León, Pedro de Alba, S/N, San Nicolás de los Garza, Nuevo León, México; 4 Centro de Investigacion en Biotecnologia y Nanotoxicologia, Facultad de Ciencias Quimicas, Universidad Autonoma de Nuevo Leon. Parque de Investigacion e Innovacion Tecnologica, Km. 10 autopista al Aeropuerto Internacional Mariano Escobedo, Apodaca, Nuevo Leon, 66629 Mexico; University of Coimbra, PORTUGAL

## Abstract

There is a current need to develop low-cost strategies to degrade and eliminate industrially used colorants discharged into the environment. Colorants discharged into natural water streams pose various threats, including: toxicity, degradation of aesthetics and inhibiting sunlight penetration into aquatic ecosystems. Dyes and colorants usually have complex aromatic molecular structures, which make them very stable and difficult to degrade and eliminate by conventional water treatment systems. The results in this work demonstrated that heavy metal-resistant *Rhodotorula mucilaginosa* strain UANL-001L isolated from the northeast region of Mexico produce an exopolysaccharide (EPS), during growth, which has colorant adsorption potential. The EPS produced was purified by precipitation and dialysis and was then physically and chemically characterized by Scanning Electron Microscopy, Fourier Transform Infrared Spectroscopy, and chemical elemental analysis. Here, the ability of the purified EPS produced to adsorb methylene blue (MB), which served as a model colorant, is studied. MB adsorption by the EPS is found to follow Langmuir Adsorption Isotherm kinetics at 25°C. Further, by calculating the Langmuir constant the adsorption capabilities of the EPS produced by the *Rhodotorula mucilaginosa* strain UANL-001L is compared to that of other adsorbents, both, microbially produced and from agroindustrial waste. The total adsorption capacity of the EPS, from the *Rhodotorula mucilaginosa* strain UANL-001L, was found to be two-fold greater than the best bioadsorbents reported in the literature. Finally, apart from determining which heavy metals stimulated EPS production in the strain, the optimal conditions of pH, heavy metal concentration, and rate of agitation of the growing culture for EPS production, was determined. The EPS reported here has the potential of aiding in the efficient removal of colorants both in water treatment plants and *in situ* in natural water streams.

## Introduction

Currently, 7x10^5^ tons of more than 100,000 commercially available dyes and colorants are produced in the world, per year, to be used in the dye, paper and pulp, textile, cosmetic and food industries. These industries use large quantities of water, which as a result generate great quantities of dye- and colorant-contaminated effluents, which in many instances are discharged into natural bodies of water [1]. Colorants discharged into the environment pose acute threats to the persistence of aquatic ecosystems [2]. Colorants are aesthetically displeasing and in many cases directly toxic to some microorganisms. Moreover, their persistence in aquatic ecosystems interferes with penetration of sunlight, which may have drastic effects on biological functions such as photosynthesis. This results in a lowering of dissolved oxygen, thereby affecting the development of higher forms of life [3]. Moreover, colorants have complex aromatic molecular structures, making them highly stable and difficult to remove [4]. Industrial colorant-containing wastewater is chemically complex since it is also likely to contain reagents from bleaching, washing, dyeing and others. Treatment of such wastewater is therefore difficult and most of the time inadequately performed by conventional wastewater treatment plants [2, 5].

Various methods have been used to remove dyes and colorants from wastewater, including coagulation and flocculation [6], ozonation [7, 8] and membrane separation [9]. Most of the time these methods only partially remove dyes; have low kinetics, high reagent/energy consumption and are economically too disadvantageous to be widely implemented [8, 10, 11]. In contrast, adsorption processes are more widely used since they are, by far, the most versatile and economically feasible [8, 12]. The most common adsorbents are aluminosilicates [13, 14]; metal hydroxides [15], and activated carbon [16]. However, bio-sorbents, such as agro-industrial waste or those microbially-produced, present a sustainable and environmentally friendly alternative. In addition, they are very cheap [17, 18] and their production does not generate toxic byproducts [17].

In nature, there are a wide variety of microorganisms that have evolved survival mechanisms to produce highly sophisticated biomolecules through elegant biological mechanisms controlled by different external stimuli (such as the presence of toxic chemicals). Among these microbially-produced biomolecules are the exopolysaccharides (EPS), biopolymers with diverse applications and structures [19, 20]. In most cases, EPS are produced when microorganisms are under conditions of stress with the mission of creating a shell-like structure that prevents toxic reagents from reaching the cell. For this reason, EPS have the ability of adsorbing a wide array of pollutants or toxic chemicals in the environment, making them potential candidates for various applications in bioremediation [21–23].

In this study, a fungus, *Rhodotorula mucilaginosa* registered as strain UANL-001L, isolated from the Pesqueria River in Nuevo León, Mexico, was shown to thrive in stressful high-metal-concentration-conditions by producing an EPS. The *Rhodotorula mucilaginosa* strain UANL-001L was therefore examined as a possible agent of bioremediation of colorants in wastewater. The EPS was characterized and its properties as a bioadsorbent were determined. Finally, the optimal conditions for its production (pH, agitation rate, and metal stress conditions) were found. The adsorption capacity of the EPS was determined by using methylene blue (MB), which is a model dye representative of the organic dyes due to its physicochemical properties and its difficulty to degrade in the environment [24]. In addition, MB is widely used in the staining of cotton, wood, and silk; and it is also highly toxic to humans [25].

## Materials and Methods

### Microorganism used

The yeast strain used in this work was isolated from the water streams of the publicly owned Pesqueria River, located in the state of Nuevo Leon, in the Northeast of Mexico. The strain used in this work was registered as UANL-001L. The Pesquería River is one of the main state rivers where, due to lax state regulations, a lot of industries release their poorly treated water effluents. Due to this situation, the Pesquería River contains different contaminants, including heavy metals. Therefore, the river was sampled (8 different 50mL samples were collected at different sites) to find possible microorganisms that are either capable of surviving or have developed mechanisms of resistance to the presence of high heavy metal concentrations. First, the different samples gathered were screened in the lab for microorganisms capable of growing in high concentrations of Pb(II). The screening was performed by plating 200 μL aliquots of the gathered samples and then incubating for 60 h. Plating was performed in YM agar plates at 28°C with increasing concentrations of Pb(II) (0 to 1000 mg metal l^-1^). Next, the microorganisms with the highest tolerance to metals were isolated. One particular yeast was isolated due to a tolerance of more than 1000 mg l^-1^ of Pb(II). Next, this isolated yeast was grown in YM plates under the same conditions described above but in the presence of increasing concentrations, from 0 to 1000 mg metal l^-1^, of different heavy metals, including: Cd(II), Pb(II), Zn(II), Ni(II), Cu(II) and Cr(VI).

The yeast was then analyzed under the microscope to determine color and Gram-staining was used to analyze its morphology.

### Biochemical profile of *Rhodotorula mucilaginosa* strain UANL-001L

The *Rhodotorula mucilaginosa* strain UANL-001L was grown on different carbon sources to determine a biochemical profile through assimilation and growth capability. The strain was grown at 28°C for 10 days in test tubes containing liquid media and the following carbon sources at 15 gL^-1^: D-arabinose, melibiose, mannose, L-Rhamnose, maltose, trehalose, aesculin, myo-Inositol, glycerol, D-Mannitol, Galactose, D-Glucitol, D-Glucose and sucrose.

To determine nitrate assimilation, the strain was grown in YM medium at 28°C, and α-naphthylamine and sulfanilic acid reagents were used [26]. The ability of the strain to hydrolyze urea was tested in Philpot’s urea agar at 28°C [26]. For both tests the results were recorded after incubation for 10 days.

### Genomic DNA extraction and 5.8S-ITS rRNA region sequencing

The yeast was identified to the *Rhodotorula* genus through morphology and biochemical assays. The fungus was cultured in YM broth at 28°C for 48h and the genomic DNA extracted extracted using the phenol-chloroform method [27]. ITS1-5.8S-ITS2 rRNA region was amplified using the universal primers ITS1 (5’—TCCGTAGGTGAACCTGCGG—3’) and ITS4 (5’—TCCTCCGCTTATTGATATGC—3’) [28].

The PCR reaction was done in a final volume of 50 μL consisting of 10 mM Tris HCl (pH 8.3), 20 mM KCl, 1.5 mM MgCl_2_, 0.2mM of each dNTPs, 1.25 Taq DNA polymerase units, both primers (ITS1 e ITS4) at 0.2 μM and 10 ng of templated DNA.

The PCR steps involved an initial denaturation at 94°C for 5 min followed by 40 cycles of denaturation at 95°C for 30 s, annealing at 60°C for 45 s, and extension at 72°C for 2 min followed by a final extension at 72°C for 7 min.

The amplicons were sent to Macrogen Inc. (Maryland,USA) for sequencing with ITS1 and ITS4 universal primers.

#### Molecular identification

The sequencing product was analyzed in the software Codoncode Aligner. Preliminarily identification was obtained using the basic local alignment search tool (BLAST) from the National Center for Biotechnology Information (http://www.ncbi.nlm.nih.gov/BLAST/).

Sequence alignment was generated using the software Mega v6.0 [29] with sequences of the 5.8-ITS rRNA region from *Rhodotorula* species already reported in the GenBank database under the following accession number: JX512682, JX512672, JX512671, JX512705, JX512677, JX494371, JX272795, JX272796, JX494370, JX494372, JX494375, JX512703 and JX512694.

A phylogenetic tree was constructed in TOPALi v2 software, [30] using a maximum likelihood algorithm, evolution model GTR+G with bootstrap analysis for 1000 replicates. The *Candida albicans* ITS sequence (accession number KP131664) was used as an external group.

### Growth of Rhodotorula mucilaginosa strain UANL-001L

All experiments were run in 500 mL Erlenmeyer flasks containing 100 mL of YM (Yeast Mold) Difco media and inoculated with an overnight yeast aliquot of 100 μL at an O.D. of 0.5. Unless indicated otherwise, all samples were grown in YM Difco Media for 106 h at 28°C, 60 rpm and a pH of 5. In order to optimize and determine how different parameters affected growth and EPS production a Response Surface Methodology (RSM) was used. The experiments were designed to vary cultivation time, orbital agitation, pH, and the addition of different metals at different concentrations (Table 1). All experiments were performed in triplicates and the standard deviations were calculated and reported.

**Table 1:** Different Levels (Low, Medium and High) of the Parameters Applied to Optimize EPS Production.

	Level		
Parameters	High	Medium	Low
**Zn(II) (g l**^**-1**^**)**	0.1	0.05	0.01
**pH**	7	5	3
**Agitation (rpm)**	120	60	0

### Separation and purification of EPS and yeast biomass

In order to qualitatively determine the rate of production of EPS and yeast biomass, *Rhodotorula mucilaginosa* strain UANL-001L was grown in 50 mL volumes, in a rotary shaker in 250 mL Erlenmeyer flasks at 60 rpm, for 60 hours in 6 different YM growth media and the control. Each medium contained one of the following transition or post-transition metals in solution as metal salts (Obtained from Fisher Sci): Cd(II), Pb(II), Zn(II), Ni(II), Cu(II) and Cr(VI) at a concentration of 0.05 g l^-1^ and a pH of 5, 2 mL samples were taken from the shaken culture of *Rhodotorula mucilaginosa* strain UANL-001L at different time points. The samples were centrifuged at 5,000 x g for 3 minutes to separate the EPS (supernatant) from the biomass (pellet). The supernatant was removed for subsequent analysis. The pellet was washed twice by resuspending it in deionized water and centrifuged at 5,000 x g for 3 minutes. The pellet was then dried in an oven at 60°C and weighed every 5 h until constant weight reading was achieved. Constant weight was usually achieved after 10 hrs.

The supernatant with the rest of the liquid was treated with ethanol in a 3:1 volume ratio in order to precipitate the EPS. The suspension of the EPS precipitate was then centrifuged to separate it from the liquid phase. After separation of the EPS, it was dissolved on bidistilled water and purified by dialysis for 24 h at 4°C with constant agitation. The dialyzed EPS was subsequently freeze-dried overnight in a lyophilizer (Labconco Freezone-6 model) and weighed. All experiments were run in triplicates and the statistical significance was reported using an ANOVA with an LSD Fisher.

### Optimization of EPS production

A Response Surface Methodology (RSM) experimental design was set up to test EPS production when four different parameters were varied. The growth conditions were the same as described before. The *Rhodotorula mucilaginosa* strain UANL-001L was initially grown 10 h and then the biomass and EPS production was recorded at different time intervals for 106 h. The parameters varied included: type of transition metal, metal concentration, pH and agitation. The EPS was obtained and weighed in the same way described previously. The contribution on EPS production was measured for each of the parameters maintaining all other constant. All of the variables tested were performed at three different levels (high, medium and low). The tests conditions included: Zn (II) concentrations between 0.01, 0.05 and 0.1 g l^-1^; pH at 3, 5 and 7; and agitation rate at 0, 60 and 120 rpm. All experiments were done in replicates of 3 and the standard deviation is reported in the results.

### Sugar reduction determination (acid-phenol method)

The total amount of carbohydrates present in the EPS samples was determined using the Acid-Phenol Dubois Method [31]. Glucose was used as the reference carbohydrate to construct a calibration curve. An EPS solution was prepared by dissolving 10 mg of dried EPS in 10 mL of distilled water. A 1 mL aliquot was then diluted with 1 mL of 80% (w/v) phenol in water and 5 mL of concentrated H_2_SO_4_ and heated at 100°C for 7 minutes. The sample was left to cool and an aliquot was diluted 1:100 in concentrated H_2_SO_4_ to measure its absorbance at 490 nm on a Varian Cary 50 UV-Vis Spectrophotometer. All experiments were done in replicates of 3 and the standard deviation is reported in the results.

### Fourier transform infrared spectra

Attenuated total reflection Fourier transform infrared (ATR-FTIR) analysis was performed on a Shimadzu IR Affinaty-1 model between 650 to 4000 cm^−1^ to identify the different functional groups present in the exopolysaccharides. These studies were carried out with each of the different EPS preparations produced under different conditions and with each of the different metals.

### Scanning electron microscopy

In order to explore the surface morphology and porosity of the EPS, micrographs were taken using a Nova NanoSEM 200 FEI scanning electron microscope with field emission.

### Chemical characterization of EPS

To detect the presence of carbonyl groups in the EPS a 2,4-Dinitrophenylhydrazine (DNPH, Brady's reagent) test was run [32]. 10 mg of the dried EPS were dissolved in 10 mL of deionized water. Two drops of this sample, 2 mL of 95% ethanol and 3 mL of Brady´s agent were added to a glass tube and all components were well mixed through vigorous agitation. The formation of a precipitate indicated a positive test for carbonyl groups [32].

In addition, a qualitative elemental analysis was performed to detect chemical groups containing nitrogen in the EPS samples. 10 mg of the dried EPS was dissolved in 10 mL of deionized water. Two drops of a Fe(NH_4_)_2_(SO_4_)_2_ saturated solution, and 2 drops of 30% KF solution were added to a 1 mL glass tube and mixed well. The solution was alkalized to a pH 9 using concentrated NaOH, was slightly heated in a water bath at 60°C for 10 seconds and finally filtered the mixture using filter paper under vacuum. Two drops of an FeCl_3_ saturated solution and enough H_2_SO_4_ was added to dissolve the Fe(OH)_3_ and the solution was acidified. Finally the sample was boiled for 30 seconds and then added aliquots of 10% H_2_SO_4_ until a greenish-blue color appeared, indicating the presence of nitrogen [33].

Finally, elemental analysis to quantify carbon, nitrogen, hydrogen, and sulfur contents was carried out using a Perkin Elmer 2400 Series II CHNS/O Elemental Analyzer. The elemental analysis was performed on each of the EPS produced after growth with exposure to each of the different metals. The reported elemental analysis is an average of the results from all of the EPS tested. The respective standard deviation is also reported.

### Determination of the biosorption capacity of EPS

Different methylene blue (MB) concentrations ranging from 0 to 3 g l^-1^ were prepared for the construction of a MB UV-vis absorbance calibration curve. Next, sets of 10 mL solutions varying from 0 to 2000 mg of methylene blue (MB) l^-1^ were prepared to perform the MB-EPS equilibrium adsorption experiments. Each solution prepared was put into contact with 5 mg of pure EPS, obtained from the growth of *Rhodotorula mucilaginosa* strain UANL-001L in YM medium for 120 hours, for two hours under constant agitation (100 rpm), at a fixed pH of 7 and 25°C. The bioadsorption curve was constructed from an analysis of the UV-Vis absorption of the methylene blue solution before and after equilibrium with the EPS, using a UV–VIS spectrophotometer (Varian Cary 50 scan) at a wavelength of 665 nm. All of the adsorption experiments were performed in triplicates and the respective standard deviations are reported.

The MB adsorption capacity for all experiments was calculated using the following equation:

$$q=\frac{V\left(C_{O}-C_{t}\right)}{M}$$

where q (mg g^-1^) is the EPS adsorption capacity; C_0_ and C_t_ (mg l^-1^) are the initial and final MB concentrations, respectively; V (L) is the volume of the solution; and M (g) is the mass of adsorbent [34].

These equilibrium adsorption data were then used to calculate the Langmuir adsorption isotherm model parameter since it is based on the premise that the maximum adsorption capacity corresponds to a saturated monolayer of adsorbate on the surface of the adsorbent. The Langmuir equation is:

$$q_{e}=\frac{q_{m}K_{L}C_{e}}{\left(1+K_{L}C_{e}\right)}$$

where *q*_*e*_ (mg g^−1^) is the MB adsorption capacity and *C*_*e*_ (mg L^−1^) is the MB concentration of the solution, both at equilibrium. The Langmuir isotherm parameters, *q*_*m*_ (mg g^−1^) and K_L_ (mg L^−1^), are constants related to the maximum adsorption capacity and the equilibrium between the adsorbed and desorbed state, respectively [34].

## Results

### Isolation of *Rhodotorula mucilaginosa* strain UANL-001L using transition metal resistance

Different 50 mL water samples were collected along the Pesqueria River in Nuevo León, México. A screening for microorganisms was then performed to identify strains capable of growing in the presence of high concentrations of metals. During the screening process a candidate strain capable of thriving in the presence of high heavy metal concentrations was found. The strain presented an MIC of 1000 mg l^-1^ to zinc and lead and an MIC between 600 and 800 mg l^-1^ to chromium (III and VI), copper, cadmium and nickel (Table 2).

**Table 2:** MICs of *Rhodotorula mucilaginosa* strain UANL-001L to Different Transition and Post-Transition Metals.

		MICs (g l^-1^)					
Isolated Microorganism	Isolation Site	Cr(VI)	Cu(II)	Cd(II)	Zn(II)	Ni(II)	PbII)
**R. mucilaginosa UANL-001L**	**Pesqueria River**	0.6	0.8	0.8	1	0.6	1

**Fig 1:**
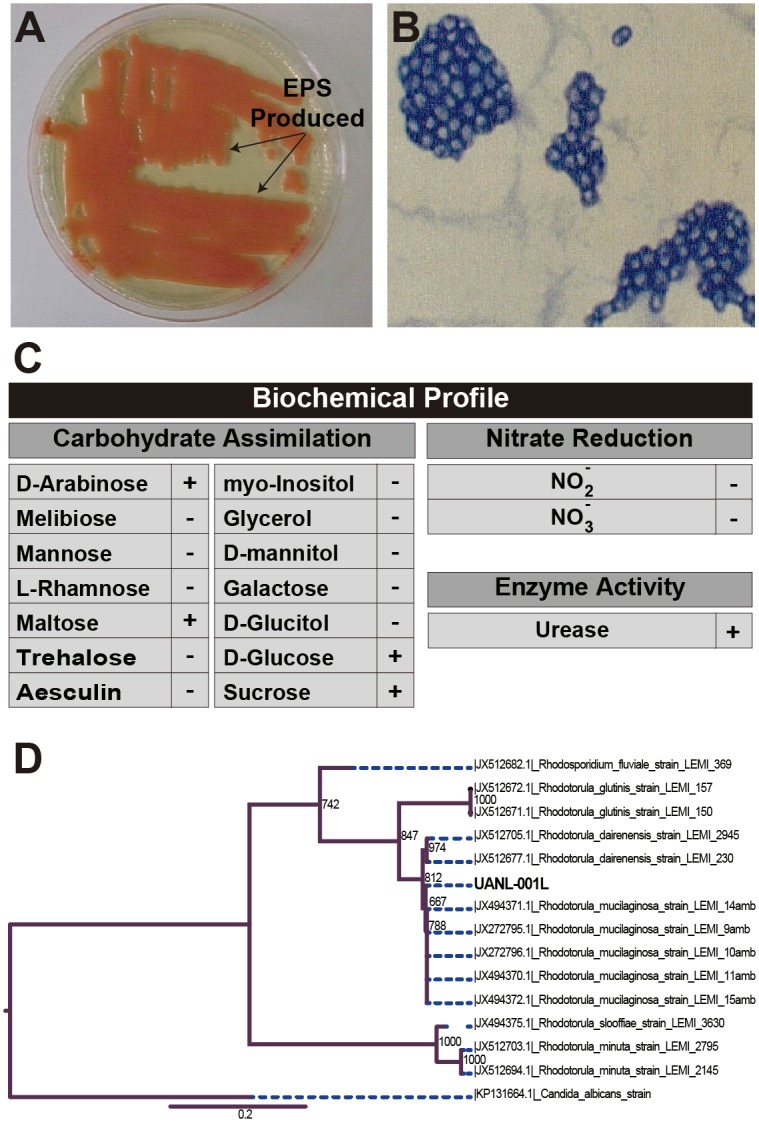
Physical Characteristics and Biomolecular Characterization of the Native Strain *Rhodotorula mucilaginosa* strain UANL-001L. **A)** Image showing pink pigmentation of the colonies. Surrounding the colonies the formation of exopolysaccharides can be appreciated as a diffused transparent slime. **B)** Microscope image at x1000 using Gram-staining and showing yeast at very dense colonies with oval shapes and without the formation of pseudohyphae. **C)** Biochemical Profile that analyzes assimilation of carbohydrates, nitrogen sources and urea. **D)** Maximum likelihood phylogenetic tree representing the phylogeny relationship between the UANL-001 strain and other rRNA ITS1–5.8S-ITS2 sequences of reported *Rhodotorula species*. The clades show reciprocal monophyly. The UANL-001 strain was grouped in the monophyletic clade corresponding to *Rhodotorula mucilaginosa* (Boostrap = 1000).

When the strain was grown in YM agar plates the individual colonies presented a salmon-pink to coral-red color (Fig 1A). Moreover, when its morphological structure was examined under the microscope, the cells were only spheroidal to oval blastoconidia with the absence of hyphae (Fig 1B). Further, a biochemical profile was performed to determine its carbohydrate, nitrogen and urea assimilation capabilities (Fig 1C). It was found that the strain was unable to grow on nitrogen sources but was able to produce urease to hydrolyze urea. Based on the morphology and the correlation of the biochemical profile with works in the literature [35], it was established that the isolate belonged to the genus *Rhodotorula*. An additional feature observed was the ability of the strain to produce considerable amounts of exopolysaccharides (EPS) at the edges of the colonies (Fig 1A).

### Molecular Identification of the *Rhodotorula sp*.

The sequencing of the ITS region was used to obtain an amplicon from the ITS1-5.8S-ITS2 region with a length of 547 bp. BLAST (http://www.ncbi.nlm.nih.gov) analysis, showed values of 99% identity with the ITS sequence of *Rhodotorula mucilaginosa* (JX494371, JX272795, JX272796, JX494370, JX494372).

The phylogenetic tree of maximum likelihood shows reciprocal monophyly corresponding to *Rhodotorula* species. As can be observed in Fig 1D, the ITS sequence of the strain and that of *R*. *mucilaginosa* share one clade cluster. Therefore, the strain was identified as *R*. *mucilaginosa* and it was registered and reported as UANL-001L.

### Transition metal induced EPS production in *Rhodotorula mucilaginosa* strain UANL-001L

**Fig 2:**
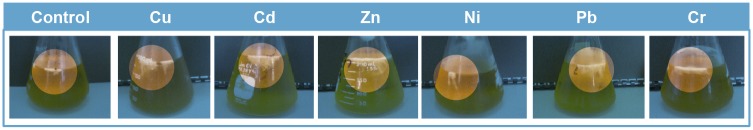
Qualitative Effect of Different Transition and Post-transition Metals in the Growth of *Rhodotorula mucilaginosa* strain UANL-001L and the Production of Exopolysaccharides. Production of exopolysaccharides is obtained when *Rhodotorula mucilaginosa* strain UANL-001L is grown in the presence of different transition and post-transition metals (Cd(II), Pb(II), Zn(II), Ni(II), Cu(II) and Cr(VI)) at concentrations of 0.05 g l^-1^.

*Rhodotorula mucilaginosa* strain UANL-001L produced different amounts of EPS when grown in YM media without metals or in the presence of different metals (Fig 2). A pattern of increased amounts of EPS produced was also noticed when the *Rhodotorula mucilaginosa* strain UANL-001L was exposed to increasing metal concentrations. It was therefore hypothesized that there could be a correlation between the presence of each of the different transition metals in the media, their concentration, and the amount of EPS produced by the strain.

**Fig 3:**
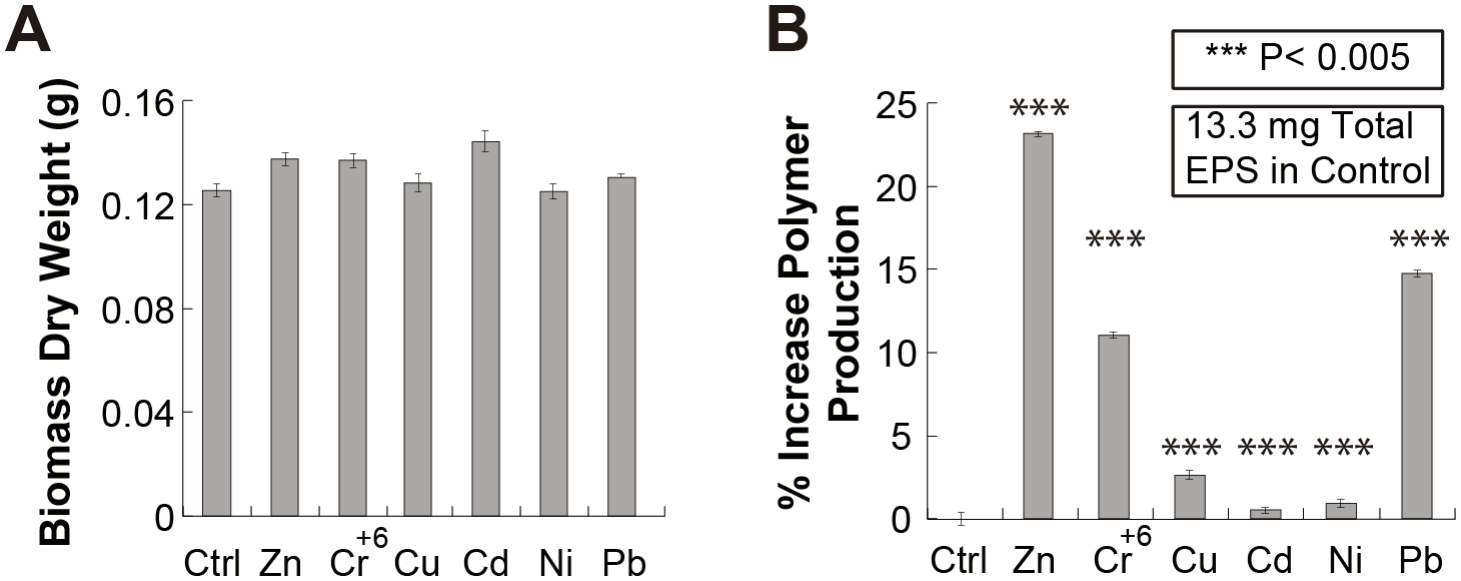
Quantitative Effect of Different Transition and Post-transition Metals in the Growth of *Rhodotorula mucilaginosa* strain UANL-001L and the Production of Exopolysaccharides. A) Dry biomass of *Rhodotorula mucilaginosa* strain UANL-001L (g) and **B)** Percentage Increase, compared to the control, in production of EPS, measured after growth in the presence of the different transition and post-transition metals (Cd(II), Pb(II), Zn(II), Ni(II), Cu(II) and Cr(VI)).

Similar amounts of dry biomass, between 120 to 150 mg, were produced in the strain when grown in the presence and absence of each of the heavy metals tested (Fig 3A). In addition, as shown in fig 3B, when comparing the different media containing metals with the control, a 25% increase in the EPS produced can be observed when 0.05 g l^-1^ Zn (II) is present. In the presence of Pb (II) and Cr (VI), between 10 and 15% EPS production increments were observed in comparison to the control. In all other cases, with the rest of the metals, less than 4% increments were observed in EPS production compared to the control, where no metal was added (Fig 3B). Ni (II) had the least effect on EPS production. Interestingly, the results show, with a statistical significance (P<0.05), that in general, the presence of these metals enhances EPS production when compared to the control.

### Characterization of EPS produced

**Fig 4:**
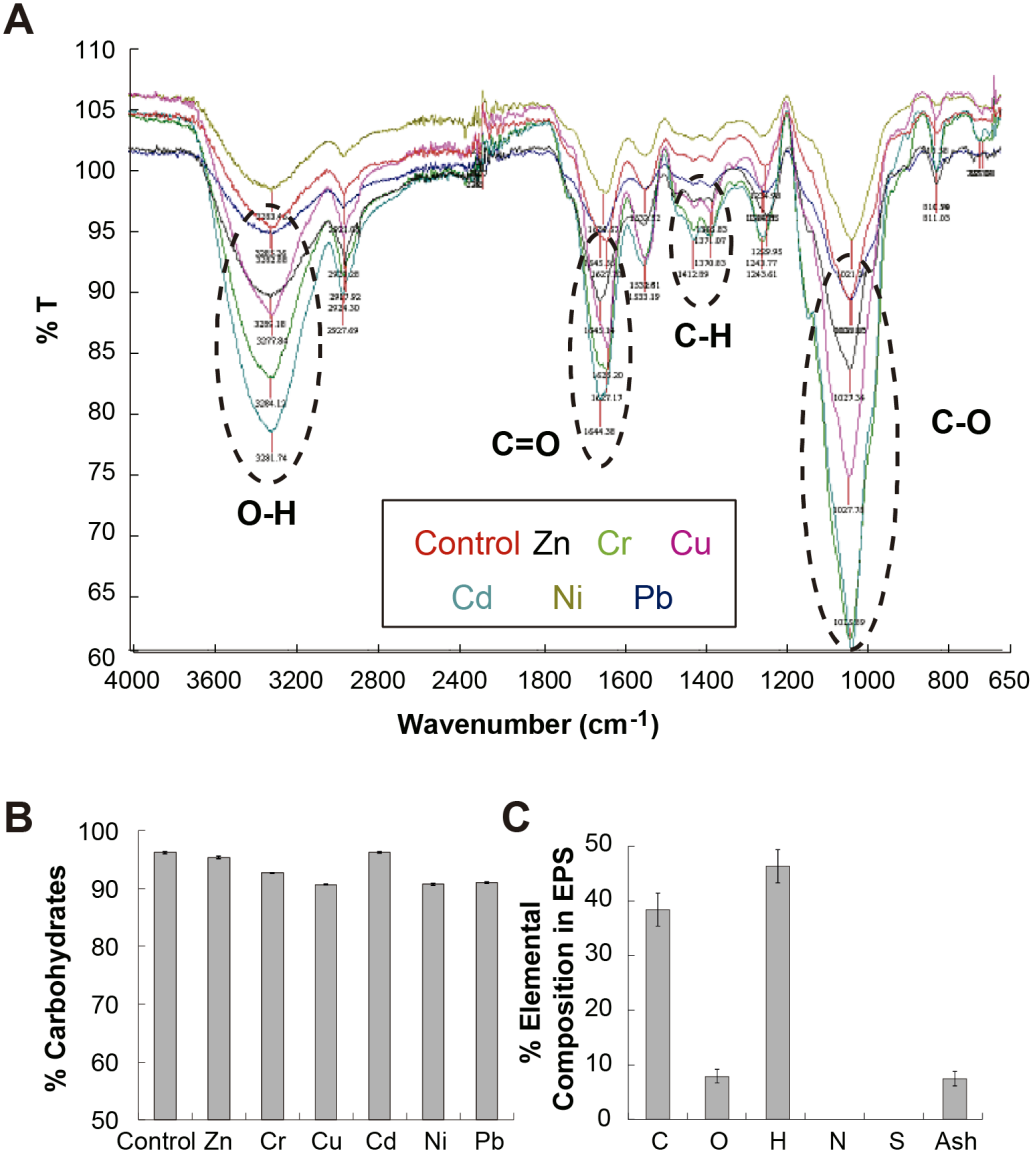
Chemical Characterization of EPS. The data show chemical analysis of the EPS produced when *Rhodotorula mucilaginosa* strain UANL-001L is grown in both the absence and the presence of the different transition and post-transition metals (Cd(II), Pb(II), Zn(II), Ni(II), Cu(II) and Cr(VI)). **A)** FTIR of the samples with the different peaks highlighted and tagged with the chemical group corresponding to each specific wavenumber. **B)** Percentage of carbohydrates present in the EPS. **C)** Average composition of carbon, oxygen, hydrogen, nitrogen, sulfur and ashes of all the exopolysaccharides produced after growth with exposure to each of the metals and the control.

FTIR analyses were ran to determine the different functional chemical groups found in each of the EPS samples (Fig 4A). The samples contained O-H groups, which appeared at 3,300 cm^-1^, peaks at around 1,644 cm^-1^ which corresponded to C = O, amino and amido groups, and peaks linked to the presence of C-O and C-H at around 1,050 and 1,450 cm^-1^ respectively [36]. To further understand the chemical structure and decipher which chemical groups contributed to the 1644 cm-1 peak, the presence of carbonyl groups in the EPS was confirmed by running a chemical assay using 2,4-dinitrofenilhydrazone [32]. Further, using chemical elemental analysis, the absence of nitrogen groups in the EPS chemical structure was corroborated. Therefore, the presence of amido or amino groups was discarded. These results confirmed that the 1644 cm-1 peak was only due to the presence of carbonyl in the samples.

Furthermore, no new peaks were observed to appear or disappear within the EPS samples, suggesting that the EPS obtained in the presence or absence of the different metals had similar chemical structures.

Afterwards, the total amounts of carbohydrates in the samples was quantified. The total percentage of carbohydrates in all of the samples (both control and in the presence of the different metals) ranged from 91 to 96% with variations of only 5% (Fig 4B). Further, using an Elemental Analyzer the average amounts of carbon, nitrogen, hydrogen and sulfur present in the samples were quantified. It was found that carbon, hydrogen and oxygen made up for 92.6% of the total samples, corresponding well with the chemical analysis and the total carbohydrate quantification in the samples (Fig 4C). The results again demonstrate the absence of N and S in the samples; confirming that amino and amido groups were absent in the EPS produced (Fig 4C).

### Morphology and adsorbent properties of EPS

**Fig 5:**
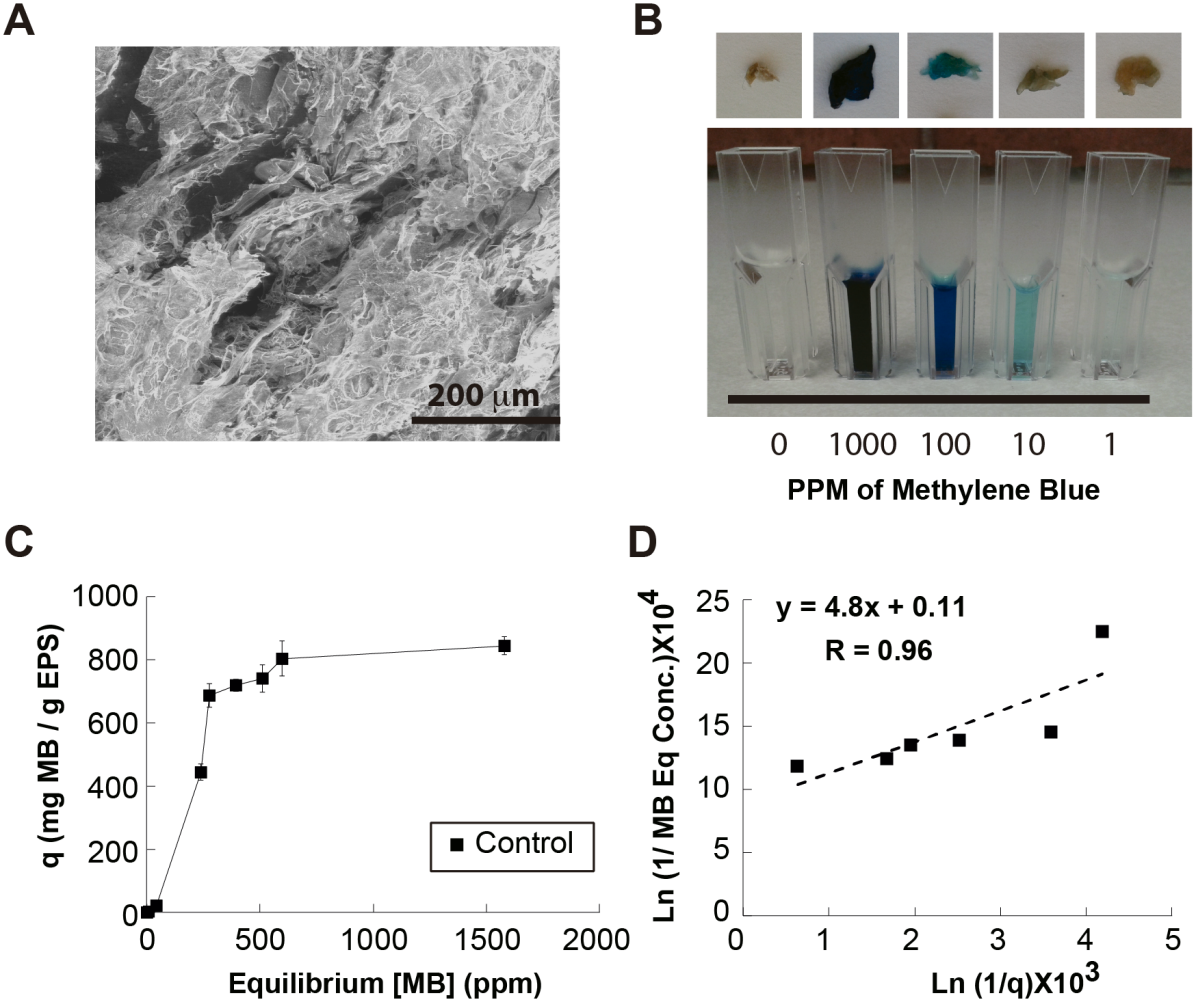
Bioadsorbent Properties of the EPS. **A)** SEM image showing morphology of EPS. **B)** Images of EPS after being stabilized for two hours with solutions of MB at different initial concentrations. **C)** Equilibrium MB concentration graphed against q (mg MB g^-1^ EPS) to obtain adsorption capacity of the EPS. **D)** Inverse of the q graphed against the inverse of the equilibrium MB concentration in solution in a logarithmic scale. The plot allows fitting to a straight line suggesting the adsorption behavior follows a Langmuir Isotherm.

EPS samples showed by SEM amorphous morphology and porous structures with high surface areas (Fig 5A). The adsorption capability of an EPS produced *Rhodotorula mucilaginosa* strain UANL-001L after growing it in YM media was determined. MB was used as a model dye to determine adsorption properties of the EPS (Fig 5B). After the EPS was exposed to solutions containing MB concentrations ranging from 0 to 2 g l^-1^, a trend can be observed which follows a Langmuir Isotherm Model with a maximum adsorption capacity around 800 mg of colorant g^-1^ of adsorbent, where the data starts to plateau (Fig 5C). Furthermore, the fit of the equilibrium adsorption data to a Langmuir Isotherm Model was analyzed, and it was found to fit such model, with a Langmuir Parameter of 4.8 (Fig 5D).

### Effects of pH, aeration by shaking and concentrations of different transition metals on optimum EPS production in flask cultures

**Fig 6:**
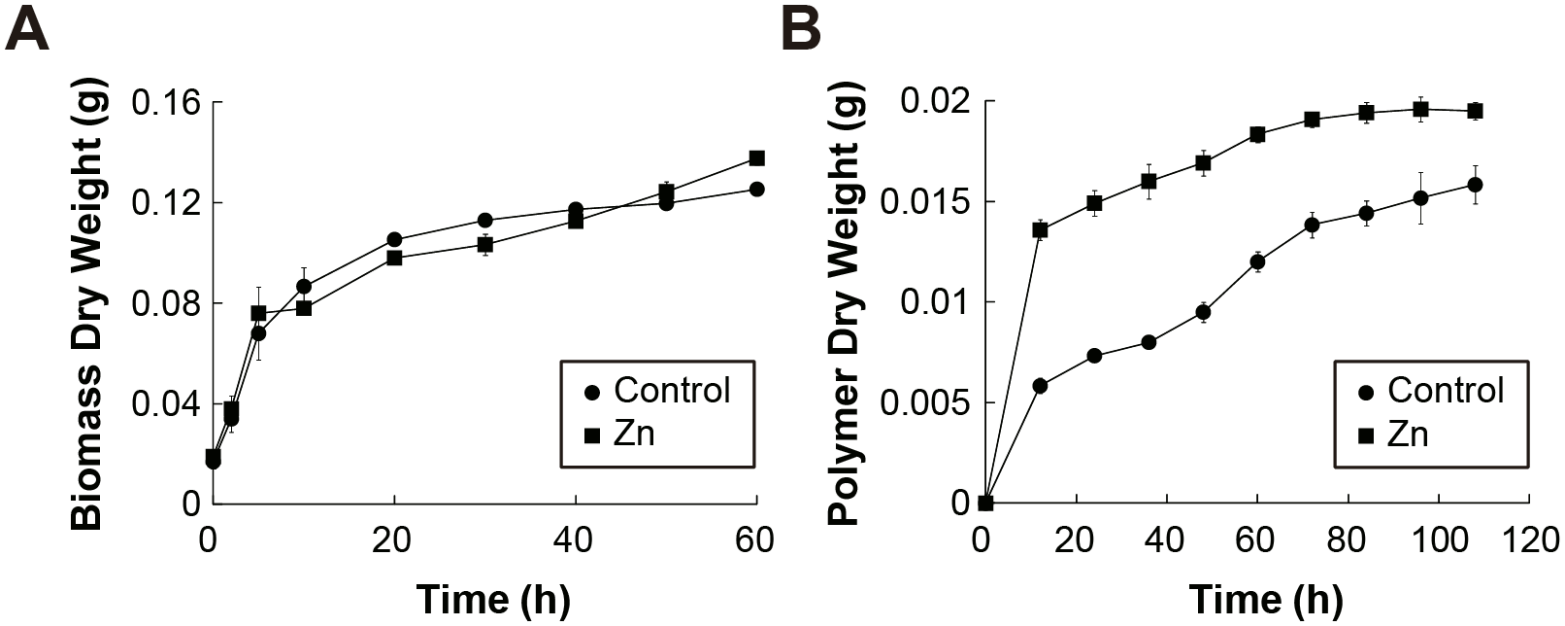
EPS Production Kinetics when Induced with Zn at 0.05 g l^-1^. The data shown represent EPS production and *Rhodotorula mucilaginosa* strain UANL-001L growth in the absence and presence of Zn at different time points. **A)** Dry biomass of *Rhodotorula mucilaginosa* strain UANL-001L (g) **B)** Dry polymer weight (g).

The kinetics of EPS production in *Rhodotorula mucilaginosa* strain UANL-001L was determined when grown in the presence and absence of Zn at 60 Rpm and pH of 5. This was done because it was found that Zn (II) is the metal, in whose presence, EPS production was stimulated the most compared to EPS production in the presence or absence of the other metals. Growth curves of *Rhodotorula mucilaginosa* strain UANL-001L in the presence and absence of added Zn (II), indicated that the same amount of biomass was formed at each time point (Fig 6A). Moreover, it should be noted that in the presence of Zn (II), *Rhodotorula mucilaginosa* strain UANL-001L strongly upregulated EPS production in the first 12 h of growth (Fig 6B).

**Fig 7:**
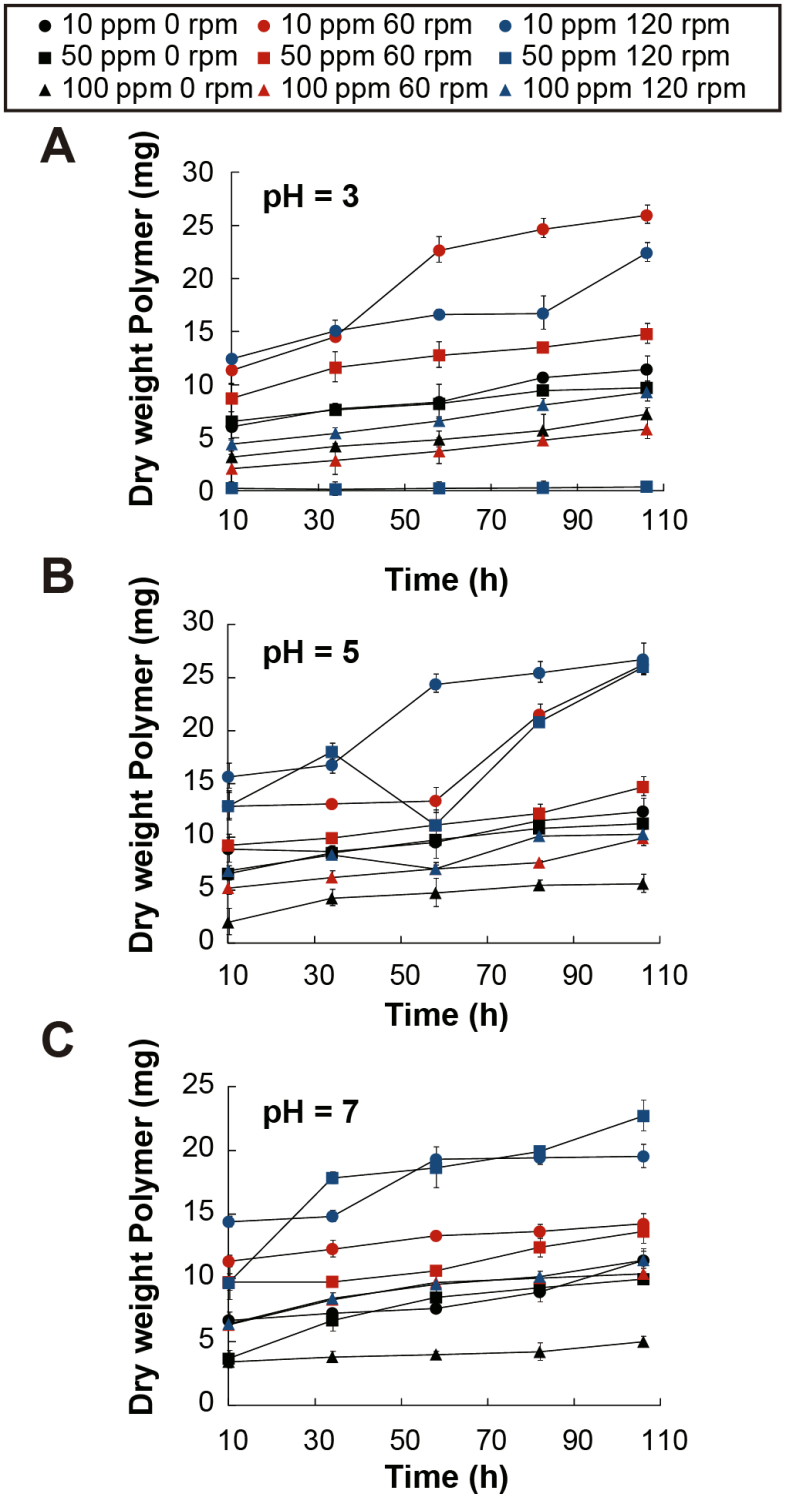
Effect of Agitation Speed, pH and Metal Concentration on Exopolysaccharide Production in *Rhodotorula mucilaginosa* strain UANL-001L. Dry weight exopolysaccharide obtained at different time points when grown at low, medium and high levels of Rpm and metal concentration and keeping pH constant at **A)** 3, **B)** 5 and **C)** 7.

To find the optimal conditions of EPS production, a Response Surface Methodology was design to study how it was affected when concentration of Zn (induction metal), pH and Rpm were individually changed between three different levels (Table 1): low, medium and high. In the first set of experiments (Fig 7A) pH was kept at 3 and agitation speed and Zn (II) concentrations were varied. The results showed that the amount of EPS obtained from the culture increased with time in all cases. The highest difference in amounts of EPS collected was achieved at a medium Rpm and a low Zn concentration. Furthermore, it was observed that at all agitation speeds the amount of EPS collected was low at high levels of Zn concentrations, suggesting that high Zn (II) concentrations impaired the production of EPS by the yeast. At pH 5, the highest quantity of EPS was produced when the agitation rate was high and the Zn concentration was either low or medium. However, at a high agitation rate and low Zn concentration, a three-fold difference in the amounts of EPS gathered between the 50 and 70 h time-points, can be observed (Fig 7B). Finally, total quantities of EPS obtained were low at medium levels of Zn and at less than a high rate of agitation, suggesting that Zn affects EPS production negatively at less acidic pHs. At low Zn concentrations similar EPS quantities were obtained, compared to the optimal conditions at pH 3, at the end of the run by agitating at medium or high levels. Finally, for the third set of experiments, the pH was increased to a neutral 7 (Fig 7C). It is clear from the results that, at a neutral pH, the most important parameter in EPS production is increased aeration (higher agitation speed). All Zn concentrations at medium and low aeration produced low amounts of EPS. These results also imply that acidic pHs allow for an increased EPS production at higher metal concentrations.

**Fig 8:**
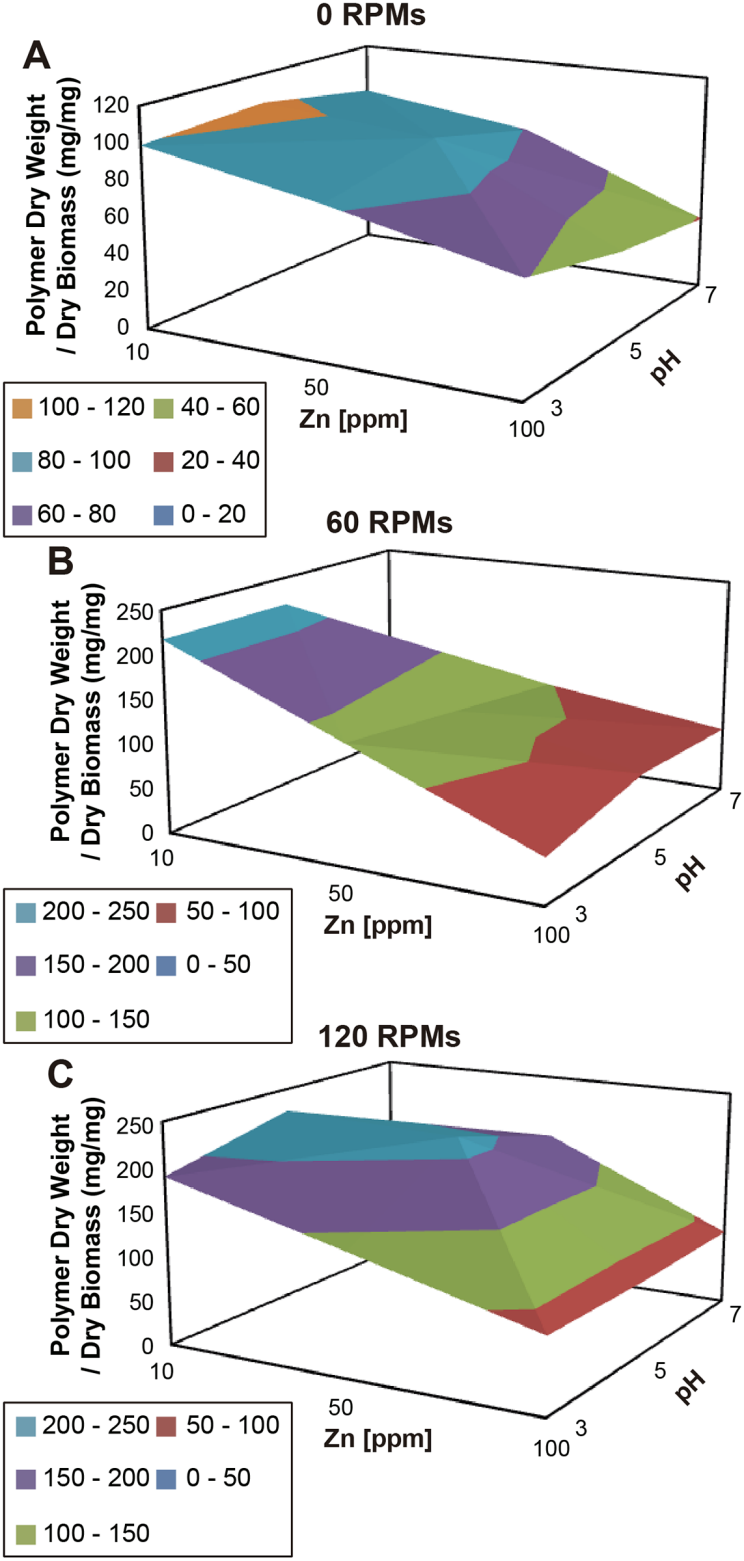
Effect of Agitation Speed, pH and Metal Concentration on Exopolysaccharide Production (mg) per Amount of Biomass (g), after growing 106 h, by *Rhodotorula mucilaginosa* strain UANL-001L. Dry weight exopolysaccharide (g) produced per amount of biomass (g) after grown for 106 h at low, medium and high levels of pH and metal concentration and keeping Rpm constant at **A)** 0, **B)** 60 and **C)** 120.

In general, for all growth conditions both the amount of biomass and EPS collected from the media does not change much after 106 h of growth. Therefore this time point was considered optimal and three-dimensional graphs were constructed by plotting time against amount of EPS produced per amount of biomass for each of the agitation speeds tested (Fig 8A–8C). At 0 Rpm (low) the optimal production is achieved at pH 5 (medium) and 0.01 g l^-1^ Zn (low) (Fig 8A). On the other hand at medium agitation speeds optimal production was achieved, with an almost 50% increase compared to low agitation speeds, at low and medium pH with low Zn concentration (Fig 8B). Finally, at high agitation speeds, the highest EPS production was obtained at medium pH and both medium and low Zn concentration (Fig 8C). However, best EPS production by *Rhodotorula mucilaginosa* strain UANL-001L occurred at pH 5, 0.01 g l^-1^ Zn, and an agitation rate of 120 rpm.

## Discussion

Microbially-produced bio-sorbents have shown to be highly efficient, very cheap and an environmentally friendly alternative to eliminate contaminants, such as colorants, from residual waters and contaminated natural water streams [17, 18]. Such is the case of microbially-produced EPS, which have been reported to be used as potent bioadsorbents [37, 38]. In this work, water samples from the Pesqueria River in Nuevo León, México were screened for microorganisms capable of producing EPS with applications in bioremediation of colorants in wastewater. This river forms part of the Bravo-Conchos hydrologic region, which includes 38.8% of the metropolitan area of the state capital, Monterrey. This river was found to be an appropriate site to explore since, due to its high water flow, it is an important collector of treated and residual waters from the principal industries of the highly industrialized sites in Nuevo Leon and its neighboring states.

The *Rhodotorula mucilaginosa* strain UANL-001L was isolated and observed to be capable of thriving in the presence of high concentrations of different transition and post-transition metals. The presence of the different metals did not affect biomass production but highly enhanced the production of EPS. These results positively correlated with the literature since production of EPS is usually a survival biological response to stress that allows microorganism to be highly resistant to different stimulus, such as changes in temperature, pH, lack of nutrients, and/or the presence of toxic compounds like heavy metals [39, 40].

After exposing the growing *Rhodotorula mucilaginosa* strain UANL-001L to different transition metals it was found that Zn (II) was the metal that stimulated the most EPS production.

Chemically, all the EPS samples contained the characteristic chemical groups found in EPS [41]. Moreover, no new peaks were observed to appear or disappear within the EPS samples produced in the presence and absence of all the different transition and post-transition metals. Further chemical analysis of the samples showed that they all had very similar carbohydrate composition and very similar percentages of the different chemical elements (C, N, O, S, H). All of these data combined suggest that the EPS obtained in the presence or absence of the different metals had similar chemical structures.

Physically, the EPS produced exhibited remarkable adsorbent properties, which can be possibly attributed to its amorphous and very porous morphology [42]. Adsorption experiments were performed using a relevant colorant, MB, since it is widely used in different industries, it can be very toxic and carcinogenic, and in human beings exposed to it can cause jaundice and cyanosis [25]. It was hypothesized that the dye would adsorb to a finite number of sites in the EPS surface, therefore a monolayer behavior was expected. The MB adsorption on EPS followed a Langmuir Isotherm Model suggesting that the maximum adsorption capacity corresponded to a saturated monolayer of MB on the surface of the EPS [34]. Moreover, the maximum adsorption capability of 800 mg colorant g^-1^ of adsorbent shown by the EPS was two-fold higher than in other microbially produced and agroindustrial waste bioadsorbents reported in the literature (q ranges between 10 to 400 mg of colorant g^-1^ of adsorbent in the literature) [34, 43–45]. Based on this, it is clear that the EPS produced by *Rhodotorula mucilaginosa* strain UANL-001L may have applications in bioremediation of colorant-containing residual waste-waters.

By using a Response Surface Methodology, production conditions were optimized through understanding EPS production behavior when parameters of Zn (II) concentration, agitation rates, and pH were changed. The yeast was found to quickly adapt to the presence of Zn (II). The *Rhodotorula mucilaginosa* strain UANL-001L up until 50 h of growth in the presence of Zn (II) triggers an exponential production of EPS, then production starts to decrease and completely stops at 106 h. Finally, it was found that for a medium pH of 5, low Zn (II) concentrations of 0.01 g l^-1^ and high aeration through elevated agitation rates of 120 rpm almost 50% increases in EPS production, compared to other conditions, could be achieved.

## Conclusions

This work reports on an autochthonous *Rhodotorula mucilaginosa* strain UANL-001L capable of surviving stress caused by the presence of heavy metal. Stimulation of exopolysaccharide formation in *Rhodotorula mucilaginosa* strain UANL-001L can be achieved by different triggers, including the presence of metals. This suggests a very interesting application in bioremediation. The physical and chemical properties of the EPS were characterized and it was found that EPS had the characteristics of a bioadsorbent. When tested for methylene blue adsorption capacity it was found that the EPS exhibited a maximum adsorption capacity of 800 mg g^-1^ of EPS, which corresponds to almost a two-fold increase when compared to other bioadsorbents reported in the literature. Moreover, the EPS is microbially produced, therefore the strain has the potential to be adapted to the use of carbon sources from agroindustrial waste, which makes it a bioadsorbent with interesting characteristics for the industry that focuses on bioremediation of colorant containing wastewaters. Finally, the optimization experiments performed in this work were able to increase EPS production up to 50% compared to the initial conditions tested. Moreover, they allowed some understanding of the strain’s mechanisms to produce EPS, paving the way to future works focusing on either production optimization or analysis of its adsorbent properties to other colorants and contaminants.
