# Correction: Metal-Induced Production of a Novel Bioadsorbent Exopolysaccharide in a Native *Rhodotorula mucilaginosa* from the Mexican Northeastern Region

**DOI:** 10.1371/journal.pone.0150522

**Published:** 2016-02-25

**Authors:** Maria Teresa Garza-Gonzalez, Daniel Barboza Perez, Augusto Vazquez Rodriguez, Domingo Ixcoatl Garcia-Gutierrez, Xristo Zarate, Maria Elena Cantú Cardenas, Ludwing Ilytch Urraca-Botello, Ulrico Javier Lopez-Chuken, Alberto Ludovico Trevino-Torres, Felipe de Jesus Cerino-Córdoba, Pavel Medina-Ruiz, Juan Francisco Villarreal-Chiu, Jose Ruben Morones-Ramirez

The first author’s name is spelled incorrectly. The correct name is: Maria Teresa Garza-Gonzalez. The correct citation is: Garza-Gonzalez MT, Barboza Perez D, Vazquez Rodriguez A, Garcia-Gutierrez DI, Zarate X, Cantú Cardenas ME, et al. (2016) Metal-Induced Production of a Novel Bioadsorbent Exopolysaccharide in a Native *Rhodotorula mucilaginosa* from the Mexican Northeastern Region. PLoS ONE 11(2): e0148430. doi:10.1371/journal.pone.0148430
